# Sex differences in human brain networks in normal and psychiatric populations from the perspective of small-world properties

**DOI:** 10.3389/fpsyt.2024.1456714

**Published:** 2024-08-22

**Authors:** Yingying Zhou, Yicheng Long

**Affiliations:** ^1^ School of Medicine, Hunan University of Chinese Medicine, Changsha, Hunan, China; ^2^ Department of Psychiatry, and National Clinical Research Center for Mental Disorders, The Second Xiangya Hospital, Central South University, Changsha, Hunan, China

**Keywords:** neuroimaging, brain network, brain connectome, sex difference, small-world network

## Abstract

Females and males are known to be different in the prevalences of multiple psychiatric disorders, while the underlying neural mechanisms are unclear. Based on non-invasive neuroimaging techniques and graph theory, many researchers have tried to use a small-world network model to elucidate sex differences in the brain. This manuscript aims to compile the related research findings from the past few years and summarize the sex differences in human brain networks in both normal and psychiatric populations from the perspective of small-world properties. We reviewed published reports examining altered small-world properties in both the functional and structural brain networks between males and females. Based on four patterns of altered small-world properties proposed: randomization, regularization, stronger small-worldization, and weaker small-worldization, we found that current results point to a significant trend toward more regularization in normal females and more randomization in normal males in functional brain networks. On the other hand, there seems to be no consensus to date on the sex differences in small-world properties of the structural brain networks in normal populations. Nevertheless, we noticed that the sample sizes in many published studies are small, and future studies with larger samples are warranted to obtain more reliable results. Moreover, the number of related studies conducted in psychiatric populations is still limited and more investigations might be needed. We anticipate that these conclusions will contribute to a deeper understanding of the sex differences in the brain, which may be also valuable for developing new methods in the treatment of psychiatric disorders.

## 1 Introduction

Previous studies have revealed that there are significant differences between the brains of males and females, which become evident in both structure and function (1–9). For instance, Cosgrove et al. (5) indicated that brain volume was greater in men than women, while women had a higher percentage of gray matter and men had a higher percentage of white matter when controlling for total volume. Moreover, global cerebral blood flow was higher in women than in men. Goldstein (6) observed that women had larger volumes relative to cerebrum size particularly in frontal and medial paralimbic cortices, and men had larger volumes relative to cerebrum size, in the frontomedial cortex, the amygdala, and the hypothalamus. Sun et al. (9) found that males had higher overall white matter (WM) fiber numbers. Gong et al. (10) found that women showed higher cortical functional connectivity (FC) mostly in the left hemisphere, whereas men had higher connectivity in the right. Published studies have shown greater local clustering in cortical anatomical networks in females as compared with males (9, 11–13). Gur et al. (7), Tunç et al. (14) and Ingalhalikar et al. (2) reported that males had greater intrahemispheric connectivity (within both hemispheres), enhanced modularity and transitivity, whereas females had higher interhemispheric connectivity and cross-module participation. Wang et al. (15) observed that significantly higher nodal efficiencies of the males were found in several brain areas of limbic and paralimbic regions, including hippocampus, parahippocampal gyrus, amygdala, and cingulated gyrus. Apart from the neuroanatomical differences, it is well‐established that sex differences in behaviors and cognitive performance have been fully demonstrated as well. Several reported research results (1, 7, 8, 16–30) have confirmed that females had advantages in language (such as reading achievement, writing abilities, and verbal fluency), episodic memory, and social cognition tasks, and males performed better on spatial processing, motor speed, and mathematical abilities. For example, Asperholm et al. (20) suggested there was a female advantage for remembering faces, odors, tastes, and colors, and a male advantage in more spatial tasks such as abstract images and routes. Furthermore, when using subnetworks that were defined over functional and behavioral domains, Tunç et al. (14) observed increased structural connectivity related to the motor, sensory, and executive function subnetworks in males. In females, subnetworks associated with social motivation, attention, and memory tasks had higher connectivity. These findings may partly explain why females and males are different in the prevalence of many psychiatric symptoms and disorders (31–33). However, the neurobiological mechanisms of these sex-based differences in the brain remain incompletely understood, which deserve further exploration in future studies.

In recent years, non-invasive neuroimaging techniques and graph theory-based network analyses have been widely used and proposed to be powerful methods for characterizing individual differences in brain structures and functions (34–38). In such a framework, the brain can be modeled as a complex network based on both structural and functional neuroimaging techniques. The widely used structural neuroimaging techniques include, for example, T1-weighted images (T1WI) (39) and diffusion-weighted imaging (DWI)/diffusion tensor imaging (DTI) (40). Functional neuroimaging techniques include functional magnetic resonance imaging (fMRI) (41), electroencephalography (EEG) (42), and magnetoencephalography (MEG) (43). Multiple topological properties of the constructed structural and functional brain networks can be then computed to reflect the changes in segregation and integration in brain systems, such as Cp (clustering coefficient), Lp (characteristic path length), γ (normalized clustering coefficient), λ (normalized characteristic path length), Eglob (global efficiency), and Eloc (local efficiency) (**Table 1**) (38, 44). Compared to traditional regions of interest (ROIs)- or voxel-based analysis, it was suggested that large-scale network analyses based on such a framework could better detect the connectivity in the brain, especially the interactions among different brain subsystems rather than a traditional regional or voxel-based analysis (35).

**Table 1:** Summary of common measures of small-world properties and their definitions.

	Measure	Full name	Definition
Measure of segregation	Cp	clustering coefficient	The probability that neighboring nodes that are also interconnected with other neighboring nodes.
	γ	normalized clustering coefficient	The normalized Cp, which is calculated for the average Cp of 100 matched random networks that preserve the same number of nodes and edges as the real network.
	Eloc	local efficiency	Eloc ensures functionally segregated processing, and measures how efficiently information is exchanged at the local level.
Measure of integration	Lp	characteristic path length	The average distance from one node to any other node in the network, expressed as the number of links that must be travelled.
	λ	normalized characteristic path length	The normalized Lp, which is calculated for the mean Lp of 100 matched random networks that preserve the same number of nodes and edges as the real network.
	Eglob	global efficiency	Eglob is a network statistic that is proportional to the average length of the shortest paths, characterizing long-range integration of the overall network.

Specially, compared with random or regular networks, the structural/functional human brain networks are thought to show an optimal balance between the segregation and integration of information processing, which is known as “small-worldness” (45–49). Generally, based on graph theory, it is known that regular networks contain many local links and are marked by a high Cp (accompanied by a higher γ and a higher Eloc) and a high Lp (accompanied by a higher γ and a lower Eglob); random networks contain many long-distance links and are marked by a low Cp and a low Lp; and small-world networks (e.g., typical brain networks) contain many local links and a few long-distance links (so-called shortcuts) and are marked by a high Cp and a low Lp. Based on the perspectives of segregation and integration, any deviation of the brain networks from the optimal small-world organizations was then thought to reflect disrupted brain structure or functioning, which can be classified into four distinct patterns: namely, randomization, regularization, stronger small-worldization and weaker small-worldization (**Table 2**) (35). Randomization, which means turning from a small-world network to a relatively random network, is characterized by at least one altered measurement of the following conditions: decreased Cp, decreased γ, decreased Eloc, decreased Lp, decreased λ, or increased Eglob. Regularization, which means turning from a small-world network to a relatively regular network, is characterized by at least one altered measurement of the following conditions: increased Cp, increased γ, increased Eloc, increased Lp, increased λ, or decreased Eglob. Stronger small-worldization, which means turning from a small-world network to a relatively stronger small-world network, is characterized by not only at least one altered measurement of the following conditions (increased Cp, increased γ, or increased Eloc) but also at least one altered measurement of the following conditions (decreased Lp, decreased λ, or increased Eglob). Weaker small-worldization, which means turning from a small-world network to a relatively weaker small-world network, is characterized by not only at least one altered measurement of the following conditions (decreased Cp, decreased γ, or decreased Eloc) but also at least one altered measurement of the following conditions (increased Lp, increased λ, or decreased Eglob).

**Table 2:** Four distinct alteration patterns from the perspective of small-world properties.

Pattern	Topological properties						
	Cp	γ	Eloc		Lp	λ	Eglob
Randomization	↓	↓	↓	And/or	↓	↓	↑
Regularization	↑	↑	↑	And/or	↑	↑	↓
Stronger small-worldization	↑	↑	↑	And	↓	↓	↑
Weaker small-worldization	↓	↓	↓	And	↑	↑	↓

↓ decreased, ↑ incerased.

Past clinical studies using various neuroimaging methods have documented that many common psychiatric disorders (e.g., schizophrenia) are associated with significant alterations in large-scale brain networks from the perspective of small-world properties. For example, Ma et al. (50) found that at rest, the patients in the schizophrenia group retained the smaller Cp, γ, and shorter path length in functional brain networks than the healthy control group, which suggested that the functional connectome in the schizophrenia group had a trend toward randomization. The majority of the other published research has also consistently demonstrated that patients with schizophrenia exhibit “more randomization” in functional brain networks (51–56). On the other hand, most research results on the functional brain networks in patients with bipolar disorder (BP) have indicated more regularization. For instance, Spielberg et al. (57) observed a trend toward regularization characterized by greater Cp and worse Eglob for the right amygdala across BP participants. Furthermore, many studies on the structural or functional brain networks in patients with major depressive disorder (MDD) have also suggested significant deviations from the optimal small-world topologies (58–60). For instance, Chen et al. (61) found that structural brain networks in MDD patients showed more regularization characterized by increased Cp, Eloc, and Lp. Overall, these findings illustrate the disparities in the pathogenesis of various psychiatric disorders from the perspective of small-world brain topology, thereby enhancing our comprehension of these psychiatric disorders.

In the field of research on possible sex differences in human brains, many researchers have also tried to use the small-world network model to elucidate differences in small-world properties of brain networks between males and females. For example, based on the fact that the hemispheric morphological networks showed small-world properties and high efficiency, the results of Choi et al. (62) indicated that brain network analysis using morphological features provided insights into the understanding of hemispheric asymmetry related to sex. Gong et al. (10) found females showed both higher overall Eglob and Eloc than males, which represented stronger cortical connectivity in females. It provided direct evidence for this hypothesis from the study of Gur et al. (63) that supposed women might make more efficient use of the available WM. Gong and his colleagues also reported that females showed greater efficiency in two well-recognized language-related regions, which might contribute to explaining the previously observed female advantage in language. Furthermore, they found males had a rightward laterality of superior parietal gyrus, which might indicate men’s advantage in visuospatial function. Additionally, Spalek et al. (64) observed that males showed higher values in brain connectivity that could point to increased functional segregation in males, which proved females had higher inter‐wiring of brain regions or a more efficient way of communication. This conclusion might provide a neural correlate for sex‐dependent memory performance differences that females performed better on the episodic memory recall because successful memory retrieval requires the conjunct activation of a network of brain regions (the less functional segregation, the higher interconnectedness) (65). However, there are still shortcomings in current research. To be specific, in several published studies on functional networks: Gong et al. (10) showed stronger small-worldization in females, Choi et al. (62) supported more regularization in males and more randomization in females, Yang et al. (1) and Yan et al. (11) observed more regularization in females, and so on. These results are not completely consistent and even conflict with each other. Therefore, it is necessary to review the previous studies to investigate whether there are consistent conclusions, while there have been no relevant reviews published in recent years to our knowledge.

To fill the gap mentioned above, this review is designed to narratively summarize the published studies on sex differences in human brain networks from the perspective of small-world properties. We aimed to compile the research findings from the past few years, focus on the investigation of sex differences in the small-world properties of both structural and functional brain networks, and discuss whether any widely accepted conclusions have been reached in this field. We incorporated all relevant structural and functional brain network studies conducted on both normal and psychiatric populations into our analysis. We anticipate that the results will contribute to a deeper understanding of sex differences in the brain.

## 2 A review of published studies

### 2.1 Searching strategy

We summarized the relevant research progress in the following paragraphs based on search results from the Web of Science (WoS). The searching strategy is as follows: “(“gender difference*” OR “sex difference*” OR “gender effect*” OR “sex effect*” OR “gender related*” OR “sex related*” OR “gender dependent*” OR “sex dependent*”) AND (“global efficiency” OR “local efficiency” OR “characteristic path length” OR “clustering coefficient”)”, and all of the reviewed articles are published before May 30th, 2024. We carefully checked the searched literature and excluded studies not conducted in humans.

### 2.2 Studies on functional brain networks in normal populations

According to the search criteria, we found a total of seven studies on sex differences in functional brain networks in normal populations, of which 3 used resting-state fMRI (rs-fMRI), 3 used EEG, and 1 used rs-MEG (**Table 3**).

**Table 3:** Summary of related studies on functional brain networks.

Reference	Sample	Neuroimaging methods	Main findings on sex differences in brain networks
(15)	10 males and 10 females (aged 21-25 years)	rs-fMRI	No significant difference in Eglob and Eloc between males and females (greater Eglob in males and greater Eloc in females)
(66)	49 older adults (age mean= 67.25y, 29 women).	rs-fMRI	More regularization in females (Increased Eloc and lower Eglob in females; higher Eglob in males)
(67)	10 males and 17 females (aged 5-18 years)	high-density rs-EEG	Alpha frequency band: no differences (low samples) but seems more regularization in males (higher Lp, Cp, σ); beta frequency band: more regularization in females (higher Cp, Lp, σ, Eglob)
(68)	24 boys and 36 girls (5.7–18.4 years)	rs-fMRI	More regularization in females (higher Lp, λ, Cp and Eloc but lower Eglob)
(69)	24 females (mean age 39) and 21 males (mean age 45)	EEG	More regularization in females’ right-hemispheric (greater right-hemispheric Eloc and lower Eglob)
(70)	220 healthy volunteers (aged 7-84 years)	rs-MEG	Similar efficiencies between both males and females
(71)	35 females and 35 males (mean age ± SD = 22.4 ± 2.3 years)	EEG	More regularization in females (higher Eloc in the delta band when focusing on the search task)

Based on the seven functional network studies, we found five data sets from seven studies were nearly consistent and reported a shift toward regularization in females compared with males (66–69, 71). Specifically, in 2013, Wu et al. (68) reported higher Lp, λ, Cp, and Eloc in females but lower Eglob than males (more regularization in females). Afterward, in 2019, Dimech et al. (66) scanned 49 older adults, including 29 women and 20 men. They found increased Eloc and lower Eglob in females (more regularization in females than males). In addition, with EEG signals Jalili (69) supported female brains showed greater Eloc and lower Eglob in the right hemisphere than male brains (more regularization in females). Aimed to explore functional network age-related changes and sex-related differences during the early lifespan, Kavčič et al. (67) studied 10 males and 17 females (aged 5-18 years) by using a high-density resting state electroencephalography (rs-EEG). They analyzed two data sets of high-density rs-EEG in healthy children and adolescents. They observed that in the beta frequency band females exhibited higher interhemispheric strength, Lp, and Cp than males (more regularization in females). Qian et al. (71) demonstrated that females showed higher Eloc in the delta band when focusing on the search task in females than males by constructing multi-frequency EEG networks (more regularization in females).

However, we noticed that the findings in several other published studies were not consistent with the above reports. Three data sets from 7 studies showed no significant difference (15, 67, 70). According to the results of Kavčič et al. (67), they analyzed EEG and connectome metrics from two different perspectives. They suggested that sex-related differences were observed mainly in the beta frequency and alpha frequency band was the most sensitive to age-related changes of the EEG-derived functional brain networks. In the alpha frequency band Kavčič et al. (67) observed that there were no significant differences but seemed more regularization in males (higher Lp, Cp, σ than females) because the data sets sample size of the test data set was very low. Besides, in the study by L. Wang and colleagues (15), they researched 20 healthy human volunteers (10 males and 10 females) and found though the females had slightly reduced global efficiencies, but slightly increased local efficiencies compared with the males, the group differences were not statistically significant. In addition, Shumbayawonda et al. (70) investigated 220 healthy volunteers. They found that using transfer entropy (TE), both males and females had similar efficiencies.

More notably, we found that the findings in several other published studies were not consistent with the above reports. For example, using rs-fMRI, Cieri et al. (72) noticed females in normal controls showed significantly lower Eglob, Eloc, and Cp, as well as significantly higher Lp (weaker small-worldization in females).

In summary, the majority of the published studies mentioned above suggest that there is a prominent trend that is more regularization in normal females and more randomization in normal males in small-world properties. However, no significant or even conflict results have been also reported in several studies. The results of these related studies are summarized in **Table 3**.

### 2.3 Studies on structural brain networks in normal populations

According to the search conditions, we found a total of nine studies on sex differences in structural brain networks in normal populations, of which 3 used T1WI and 6 used DWI/DTI (**Table 4**).

**Table 4:** Summary of related studies on structural brain networks.

Reference	Sample	Neuroimaging methods	Main findings on sex differences in brain networks
(10)	47 males and 48 females (aged 19–85 years)	DTI	Stronger small-worldization in females (higher Eglob and Eloc)
(1)	A healthy sample of 28,821 from UKBB (15,073 females, 13,748 males)	(based on cortical thickness) T1WI	More randomization in males (higher Eglob)
(9)	Baseline: 28 females (aged 18-25 years) and 43 males (aged 22-53y) Longitudinal: 15 females (aged 26-61 years) and 13 males (aged 29-53y)	DTI	Stronger small-worldization in males (greater Eglob, lower Lp and increased Cp); weaker small-worldization in females (higher Lp and decreased Cp)
(64)	264 males and 391 females (aged 18-35 years)	DWI	More randomization in females (lower Cp)
(62)	150 females and 135 males (aged 22-36 years)	(based on cortical thickness) T1WI	More regularization in males (greater Eloc and lower Eglob in the left hemispheric network); more randomized in females (greater Eglob and lower Eloc in the left hemispheric network)
(8)	111 females and 61 males (aged 20-65 years)	(based on cortical volume) T1WI	More randomization in females (higher Eglob of structural covariance networks)
(73)	99 children (54% boys, aged 6-11 years)	DTI	No significant differences in the structural connectivity and global network properties between male and female children
(11)	38 females (aged 18-24 years) and 35 males (aged 18-27y)	DTI	More regularization in females (greater Eloc)
(74)	310 twins and their older siblings (158 boys and 172 girls but 20 of them with poor quality DTI scan); mean ages: 10, 13, and 18 years	DTI	No significant sex differences

Based on the nine structural network studies, we found that results from 9 studies showed different conclusions in small world properties between males and females. These studies include 1 report of stronger small-worldization in females (10), 1 report of stronger small-worldization in males and weaker small-worldization in females (9), 1 report of more regularization in males and more randomization in females (62), 2 report of more randomization in females (8, 64), 1 report of more randomization in males (1), 1 report of more regularization in females (11), and 2 reports of no significant differences (73, 74).

In 2009, Gong and colleagues (10) recruited 47 males and 48 females and found that women showed greater overall cortical connectivity both locally and globally as well as lower integrated cost compared with men, which meant women had higher Eglob and lower Eloc (stronger small-worldization in females).

However, we noticed that there were some different findings as follows. For example, using the DTI technique and graph theory methods, Sun et al. (9) in 2015 reported a more economical small-world architecture in females regardless of scan time point. To be specific, males showed greater Eglob, lower Lp, and increased Cp (stronger small-worldization in males) as well as females showed higher Lp and decreased Cp (weaker small-worldization in females).

Afterward, in another study, using the T1-weighted magnetic resonance imaging scans of 150 females and 135 males, Choi et al. (62) studied a cortical thickness-based brain structural covariance network named hemispheric morphological network and supported males showed greater Eloc and lower Eglob in the left hemispheric network (more regularization in males), while females showed greater Eglob and lower Eloc in the left hemispheric network (more randomization in females). Moreover, Spalek et al. (64) observed that males showed higher values in weighted transitivity on node level (higher Cp) and increased segregation compared to females (more randomization in females). Additionally, using surface-based morphometry and structural covariance (SC) analysis, Shi et al. (8) constructed structural covariance networks (SCN) based on cortical volume. They found that females had a higher number of SC connections which meant superior network integration, and high Eglob of SCN compared with males (more randomization in females).

There are other studies that reported opposite conclusions as well. For instance, Yang et al. (1) suggested that males showed higher nodal strengths throughout the brain, greater global and local structural covariance as well as higher Eglob (more randomization in males). On the other hand, by constructing weighted cortical networks from 72 young healthy participants (including 38 females and 35 males), Yan et al. (11) found that females had greater Eloc than males (more regularization in females). They also found smaller brains showed higher Eloc in females but not in males.

Furthermore, Kim et al. (73) found that there were no significant differences in the structural connectivity and global network properties between boys and girls. Koenis et al. (74) reported that adolescents with higher intelligence had higher Eglob and Eloc but there were no significant differences between boys and girls at each time point separately for FA-weighted global and local efficiency. They concluded the associations between global and local efficiency of the brain with intelligence revealed no evidence for quantitative or qualitative genetic sex differences.

In summary, we observed several different conclusions in the aforementioned studies of structural networks in normal populations. In conclusion, there seems to be no consensus to date on the sex differences in small-world properties of the structural brain networks in normal populations. The results of these related studies are summarized in **Table 4**.

### 2.4 Studies conducted in psychiatric populations

There were several studies that have investigated the sex differences in clinical populations with several common psychiatric disorders (e.g., participants with depression and substance addiction). However, we noticed that the number of related studies was much less than those conducted in normal populations. The details of these studies are briefly listed as follows.

Cieri et al. (72) applied graph theory analysis on rs-fMRI data to evaluate sex differences of brain functional topography in Alzheimer’s Disease (AD) patients, early mild cognitive impairment (eMCI), and normal controls (NCs). They found that NC females had significantly lower Eglob, Eloc, and Cp, but higher Lp compared to NC males (weaker small-worldization in healthy females). This sex difference diminished in eMCI, though females continued to show weaker small-worldization than males. And no significant sex difference was observed in AD for graph theory metrics. They indicated that NC females showed a pattern more similar to the pathological groups in all graph theory metrics and worse integration and segregation values compared to men, despite significantly better verbal learning scores, which were partially consistent with work showing higher modularity and transitivity in young men versus women (2) while not consistent with recent review in children and young adult (7). These results might suggest that weaknesses in segregation and integration contribute to vulnerability of women to AD. In addition, they observed NC and eMCI females had better learning performances than males, confirming previous findings that females retained an advantage in verbal learning and memory at least before significant levels of impairment within a sample of older adults (75).

Using diffusion-weighted MRI in *de novo* Parkinson’s disease (PD) patients without medication (149 males, 83 females) and 117 healthy controls (78 males, 39 females), Tremblay et al. (76) measured structural brain differences between sexes in PD. They indicated that males with PD showed significantly lower Eglob and Eloc compared to females with PD (weaker small-worldization in males with PD). They identified that with PD overall had more regional atrophy than females, mostly in cortical regions. Atrophy of the regions showing disrupted Eloc in PD had been reported (77, 78), along with a significant relationship with cognitive decline (78, 79). Male sex had been shown to be a contributor to brain atrophy and clinical severity in PD in PPMI data (78). This was in line with previous studies on PD reporting worse prognosis in males compared to females (80), faster development of difficulties in activities of daily living (81), and a higher risk of developing cognitive impairment (81, 82). For males with PD, positive relationships were found between Eloc and two measures of memory, including correlation between the Eloc of the left inferior frontal gyrus and episodic memory (83, 84), and between the Eloc of the right insula and working memory scores (85, 86). Disruption in the Eloc of the structural connectivity of these brain regions might impact episodic memory and working memory.

Qiu et al. (87) scanned 56 tobacco use disorder (TUD) participants (25 females) and 66 non-TUD participants (28 females) by using rs-fMRI. They found that TUD participants had significantly lower Eglob and a lower trend of Eloc than non-TUD participants in males (weaker small-worldization in TUD males than non-TUD participants) while there was no significant difference of Eglob or Eloc in females, which indicated that men and women TUD participants had different responses to tobacco. Their results were consistent with previous reports that heavy smoking adults showed lower Eglob (88), indicating information transfer within cliquishness was much slower in males. Other studies reported that smoking males experienced faster cognitive decline in global cognition and executive function compared with nonsmoking adults (89), and males were more susceptible to smoking than females (90). They found that compared to non-TUD participants, the connections between VS and EC as well as SC were lower in men but not in women TUD participants. The lower intermodular connections between VS and EC might indicate the impairment of inhibitory control ability in smoking men. The lower intermodular connections between VS and SC might be related to the processing disorder of visual-related information during chronic addiction in men. These results provided new insights into sex-related differences among TUD participants in terms of Eblob and Eloc.

Whether there are significant sex-related effects on the small-world brain network properties was not reported in most of the other studies. For example, Peng et al. (91) found significant differences in the small-world brain network properties between the post-stroke depression (PSD) and the non-PSD group. The brain network characteristics could reflect the severity of PSD to some degree and might provide new insights into the understanding of PSD and new methods for the diagnosis of PSD. The brain functional network analysis might potentially be to help with the early diagnosis of brain diseases in the near future (92). Nevertheless, sex was only treated as a confounding factor, and they didn’t report whether there were significant sex effects on the small-world network properties.

In summary, limited number of studies reviewed in psychiatric populations from the perspective of small-world properties and more studies conducted in psychiatric populations may be warranted to obtain more valuable results.

## 3 Discussion

### 3.1 Main findings

This manuscript aims to summarize published findings on the sex differences in structural and functional human brain networks from the perspective of small-world properties. Based on 4 patterns of altered small-world properties proposed by Suo et al. (35): randomization, regularization, stronger small-worldization, and weaker small-worldization, the most prominent trend in the functional network studies reviewed here is more regularization in normal females and more randomization in normal males. However, it seems that no consistent alterations were reported in structural brain networks in normal populations. Moreover, we noticed that the number of related studies conducted in psychiatric populations is still limited, and more investigations in clinical populations might be needed.

### 3.2 Sex differences in functional brain network in normal populations

Large proportions of the reported neuroimaging techniques were rs-fMRI and EEG. Of the 3 studies using rs-fMRI, 2 studies consistently reported more regularization in females (66, 68), 1 study reported more randomization in males (68), and 1 study reported no significant difference (15) between mate and female while characterized by a litter greater Eglob in male and a little greater Eloc in female, which showed a trend toward a litter more regularization in females and more randomization in males. The conclusions of studies using rs-fMRI are almost not contradictory. Of the 3 studies using EEG, 3 studies consistently reported more regularization in females (67, 69, 71), 1 study reported more randomization in males (69), and 1 study reported no difference but seemed a little more regularization in males (67).

The global efficiency of a network can be conceptualized as the efficiency of parallel information transfer in the network (93), which can be thought to be a more robust measure of integration. High global efficiency reflects effective interactions or rapid transfers of information between and across remote cortical regions that are believed to form the basis of cognitive processes (10). In the functional network studies, four studies reported similar conclusions, which showed a shift toward higher Eglob in males (15, 66, 68–70). Among these above, Wu et al. (68), Jalili (69) and L. Wang et al. (15) showed higher Eglob in males than females. Dimech et al. (66) reported males had higher Eglob but they also found Eglob was negatively associated with cardiorespiratory fitness (CRF) in the default, frontoparietal control, and cingulo-opercular networks only in males. However, there is one study that reported an inconsistent conclusion that females’ Eglob was higher than males. Specifically, Kavčič et al. (67) found females showed higher Eglob compared to males in the beta frequency band.

The local efficiency is a measure of communication between nodes (94), which can be thought to be a more robust measure of segregation. High local efficiency implies modularized information processing among nearby regions (10). Five studies also showed similar results: both observed females showed higher Eloc than males (15, 66, 68, 69, 71). For instance, Wu et al. (68) and L. Wang et al. (15) showed higher Eloc in females than males. Dimech et al. (66) found Eloc was positively associated with CRF in the default, frontoparietal control, and cingulo-opercular networks, which was more robust in male versus female older adults. Jalili (69) found females showed significantly greater right-hemispheric local connectivity (Eloc) than males. Qian et al. (71) also noticed that females showed higher Eloc in the delta band when focusing on the search task in females compared to males.

Overall, the most prominent trend in the functional brain network studies about topological properties is a shift toward more randomization in normal males, and a shift toward more regularization in normal females.

### 3.3 Sex differences in structural brain network in normal populations

In the structural network studies, large proportions of the reported structural neuroimaging techniques were DTI/DWI and T1WI. Of the 6 studies using DTI/DWI, 2 studies consistently reported no significant differences between different genders (73, 74), 1 report of more randomization in females (64), 1 study reported stronger small-worldization in males and weaker small-worldization in females (9), 1 report of stronger small-worldization in females (10), and 1 study reported more regularization in females (11).

We observe the inconsistencies in the above studies using DTI/DWI. Based on the same imaging techniques, we also infer that the reasons for different findings may be a consequence of different image acquisition methods, analytical methods, different designs of studies, different age distributions and sample size. First, the image acquisition of these researches used different parameters. Second, Spalek et al. (64) observed that males showed higher values in weighted transitivity that on node-level referred to as Cp (increased segregation) and females showed higher interconnectedness, which were in line with previous research that a cross-module function in female’s brains and a more modular function in males (2, 95, 96). These effects were mostly pronounced in regions of the reward circuitry (97, 98), in a region involved in visual encoding (99) and in the somatosensory and motor cortices (100). Furthermore, they found weighted transitivity had a negative association with memory performance of positive pictures that females had a pronounced memory advantage than males when analyzing the memory performance of positive pictures, which confirmed female’s advantage in episodic memory tasks compared to males. Compared to the research of it, Sun et al. (9) adopted more network metrics to characterize the global topological organization of structural brain networks. They found a predilection for global information integration and more globally efficient for information transfer in males, which might be attributed to the strengthened bilateral intra-hemispheric connections. Both Sun et al. (9) and Gong et al. (10) found females tended to be more economical in small-world architecture. The study of Gong et al. (10) found females showed greater interhemispheric connectivity and possibly accounting for the more bilateral pattern in language-related activation of women (101). Yan et al. (11) found that females had greater local efficiencies than males in their cortical anatomical networks. The possible explanations might be that the brain size effect on local efficiency is significant in females but not in males (11), and that brain size had different effects on the morphologies of anatomical structures between males and females (63, 102, 103) For example, women had a larger corpus callosum (102), which suggested greater interhemispheric connectivity (10). Third, Sun et al. (9) designed not only baseline study but also longitudinal follow-ups. Gong et al. (10) only used cross-sectional data therefore could be influenced by potential cohort effects. Fourthly, the weighted transitivity (Cp) was negatively correlated with age in a cohort of healthy young adults (64). There were also changes in the underlying network organization that resulted in decreased local efficiency with age (10). Meanwhile, the age range of some participants for study were larger (9, 10), which might affect the results. Finally, we noticed that the sample sizes in some studies are relatively small, which might lead to less reliable results.

Of the 3 studies using T1WI, 2 studies consistently reported more randomization in females (8, 62), 1 study reported more randomization in males (1), and 1 study reported more regularization in males (62). In detail, Yang et al. (1) reported that males had higher global efficiency than females, whereas Choi et al. (62) found females were more globally efficient in the left hemispheric network. The above two studies used the same methodology. We speculate that the reasons for different findings may be a consequence of sample size, different age groups, and the selection of the brain atlas.

In summary, in the structural network studies, there are three studies that reported similar conclusions, which showed a shift toward higher Eglob in females relative to males (8, 10, 62). Among these above, Gong et al. (10), Choi et al. (62), and Shi et al. (8) showed higher Eglob in females than males.

These results are not consistent and even conflict with those studies mentioned in the last paragraph. Two studies reported similar conclusions. Sun et al. (9) showed males exhibited higher Eglob suggesting a predilection for global information integration which might be attributed to the strengthened bilateral intra-hemispheric connections. Yang et al. (1) found that males showed higher Eglob, as well as higher regional covariance (nodal strengths) in both hemispheres compared with females.

Two studies found that females showed higher Eloc than males (10, 11). In detail, Yan et al. (11) found that females had greater Eloc than males and smaller brains showed higher Eloc in females but not in males. There was also another published finding that supported a similar conclusion. Lou et al. (104) found that phonemic decoding was also positively correlated with the Eloc of the reading network that was significantly relevant in girls, but no significant correlations were found in the boys group. And the phonemic decoding subtest is related to word reading efficiency. Another study by Choi et al. (62) noticed that males showed higher Eloc than females.

Overall, there seems to be no consensus to date on the sex differences in small-world properties of the structural brain networks in normal populations.

### 3.4 Possible reasons for inconsistent findings

Here, we propose that the inconsistencies in previous studies (especially on structural networks) may be partly due to several reasons: sample size, different age groups, methodology alterations, and so on.

First, we noticed that the sample sizes in some studies are relatively small, which might lead to less reliable results (15, 67). The test data set sample size was much smaller, therefore higher probabilities were less likely to be observed. Kavčič et al. (67) supported that they couldn’t claim that any significant differences in graph metrics existed between sexes in the alpha frequency band in either of the data sets sample size of the test data set was very low (the subgroup of only 10 males), therefore these results should be interpreted with caution. As a result of the small sample size, we probably could not detect sex differences, which were observed in a larger validation data set. In addition, the number of the participants who completed the longitudinal scan is relatively small and future research with an independent larger study sample over a longer time frame is needed to confirm the observations (9).

Second, the sex differences in human brain networks of small-world properties may be associated with age. In the systematic review of Richmond et al. (105), they summarized that for SC, diffusion MRI findings indicated decreased clustering, and increased Eglob and Eloc with age from the prenatal to late adolescent period (stronger small-worldization with age). The clustering coefficient, which is related to weighted transitivity (38) showed a decrease in younger age groups but an increase in older ages (106). There was also some indication pointing to the development tendency toward a less economical topology with aging and there was a significant gender-time interaction on Cp. Specifically, males showed an insignificant increase in Cp whereas females exhibited a significant decrease (9). However, Gong et al. (10) found that the aging network became less connected (cost more), overall cortical connectivity became reduced, and the disruption of anatomical connectivity in aging might impair the functional integration between areas. Another study reported consistent results that network efficiency of functional networks reduced in normal aging using rs-fMRI techniques (107). There is another study that reported diverse conclusions. Henry et al. (108) found that Eglob had an opposite relationship with age by first decreasing and then increasing in the autism spectrum disorder (ASD) group. So, diseases could affect brain network topological properties.

Third, different methodology alterations may lead to different results. Sun et al. (9) and Yan et al. (11) used the DTI deterministic tractography method to reconstruct structural brain networks. Despite being widely used, this method has a limited capacity for resolving the fiber crossing issue and may result in a loss of the estimated fibers (109). The probabilistic tractography used in the study of Gong et al. (10) had showed advantages in tracking specific WM tracts relating to fiber crossing and it outperformed the deterministic method in overcoming the fiber crossings and robustness to the image noise (110–112). Therefore, further attempts could be conducted on structural brain networks reconstructed by probabilistic diffusion tractography methods (2, 12). However, it remained possible to miss some biological connections or included spurious connections in the cortical network even after the thresholding procedure. To control the influences of the total fiber number differences across subjects and investigate the salient topological differences between males and females beyond the simple gender-related differences in WM connectivity strength, Sun et al. (9) performed a normalization approach prior to the network metrics estimation (113). However, in weighted network analyses which incorporated the variations in the strength of connectivity into the network metrics estimation, significant gender effect in the WM microstructure might have some potentially pronounced effect on the network measures. It would therefore be important for future attempts to explore the progressive gender differences of structural brain networks with different weighting approaches (110). Furthermore, recent studies have suggested that the node definition by different parcellation scales might result in different properties of brain networks so that graph analyses with different spatial resolution is encouraged in the future to provide more comprehensive information on the gender related topological differences of structural brain networks (114–116). We also speculate that the apparent inconsistencies might have something to do with the different designs of studies (longitudinal *vs*. cross-sectional) and most of the current studies mentioned were cross-sectional designs, which might result in some differences in diagnosis. For example, Gong et al. (10) only used cross-sectional data therefore could be influenced by potential cohort effects. Based on DWI tractography (64), a one-to-one relationship between a given diffusion parameter and the underlying tissue structure was not possible Inherent EEG limitations for studying functional connectivity should also be considered (67).

Another limitation in the study of Shumbayawonda et al. (70) is the use of unbalanced numbers of subjects in groups. We also speculate that the apparent inconsistencies might be due to the different designs of studies. In addition, the sex differences in human brain networks of small-world properties may be associated with individual differences or psychological quality. From graph-based metrics, Wang et al. (117) detected significantly greater Eglob and Eloc but shorter Lp in the anatomical networks of the world-class gymnasts as compared to healthy age and sex-matched students, which showed champions had stronger small-worldization. Furthermore, most of the current studies mentioned were cross-sectional designs, which may result in some differences in diagnosis. Future studies can also benefit from longitudinal follow-ups. Further studies are needed to reconcile the apparent inconsistencies and confirm our findings.

### 3.5 Limited number of studies conducted in psychiatric populations

We found that the number of studies was limited to sex differences in small-world properties of brain networks in psychiatric populations. For example, Cieri et al. (72) observed that females with eMCI had significantly lower Eglob, Eloc, and Cp, but higher Lp than males with eMCI (weaker small-worldization in females with eMCI), while no significant sex differences in AD. Tremblay et al. (76) reported that males with PD showed significantly lower Eglob and Eloc compared to females with PD (weaker small-worldization in males with PD). Qiu et al. (87) found that TUD participants had significantly lower Eglob and a lower trend of Eloc than non-TUD participants in males (weaker small-worldization in TUD males than non-TUD male participants), while there was no significant difference between TUD and non-TUD subjects in females. However, for most of these, the sample size was relatively small and there were no repeated studies. This may be due to that in most published neuroimaging studies on psychiatric disorders, sex was only treated as a confounding factor (59, 118, 119), and possible sex-related effects were not heeded.

Cieri et al. (72) showed that neuroaging seemed to occur earlier in females and pathological biomarker changes such as FC seemed to anticipate the cognitive impairment observed in AD. They confirmed differences between individuals with healthy and impaired cognition and showed new differences between males and females. Moreover, the default network is “normally” highly clustered, but it tends to lose “connectedness” in neurodegeneration becoming more intermingled with task positive networks (120). Therefore, more studies to analyze the specific networks is needed.

In the study of populations with PD, the sex differences in Eglob were attributable to differential sex effects of aging but not to PD, as the association disappeared when they used W-scores corrected for sex. While the results of decreased Eloc in males were attributable to PD. It is possible that the greater disruption in connectivity in males seen here results from a generally faster neurodegenerative process. Future studies with the longitudinal PPMI dataset should address this issue. In sum, a neuroprotective effect of estrogen and sex differences in dysregulation of gene expression might underpin the existence of sex differences in PD.

In the study of TUD, these results might provide a new reference for exploring the potential neural mechanisms of sex differences in TUD and lay the foundation for the specific strategy of prevention and treatment of TUD in males and females. However, the differences could be affected by age-related cortical differences with sex (121), education distribution, smoking status, other addictions, menstrual cycle phase, and level of gonadal hormones. These factors could not be ignored in future research.

Therefore, more investigations might be needed to confirm these conclusions in psychiatric populations, which might help improve our understanding of the underlying pathogenesis of psychiatric disorders to help to tailor individualized interventions and provide sex-sensitive treatment.

### 3.6 Future perspectives

As discussed above, future studies can be performed to obtain more reliable results on sex differences in human brain networks in normal populations from the perspective of small-world properties, by offering larger sample sizes, more age groups, longitudinal designs, and the use of standardized methodologies and so on. This is especially necessary for structural brain networks as there seem to be no consensus conclusions in published studies to date.

In the future, more studies conducted on psychiatric disorders may be also needed to obtain more valuable results. In these studies, researchers can pay more attention to sex effects to understand the sex-related heterogeneity of psychiatric disorders, rather than only treat sex as a confounding variable. There are results showed substantial disruptions mainly in Eloc and Eglob of the structural connectivity in PD (76, 122). Further investigations should be done to verify the existence of sex differences using other metrics and future studies should investigate whether the sex differences change over time.

Another valuable future direction may be to investigate the possible sex differences in the dynamic functional brain networks. In recent years, research on dynamic fluctuations of the functional brain network organizations so-called “dynamic functional brain network” has emerged (123–125). Especially, some prior studies have promoted the “temporal small-world/dynamic small-world” model under such a framework, which could capture important information ignored by traditional “static” brain network model (126–128). However, little is known about the possible sex differences in brain networks from the perspective of “dynamic” small-world properties, which deserves further investigation.

### 3.7 Limitations

This study has several limitations. First, the differences in preprocessing steps and analytical methods can produce inconsistent results, and thus the accurate integration of results across different studies is difficult. Second, some published studies might have been missed during the literature search because of the chosen keywords. Finally, the current knowledge is much limited on possible relationships between the sex differences in human brain networks from the perspectives of four patterns of altered small-world properties and several disorders. The number of studies is still limited to our knowledge, and more studies may be needed in the future to investigate the sex differences in human brain networks from the perspective of small-world properties.

## 4 Conclusions

In summary, this manuscript reviewed the published studies regarding the possible sex differences in human brain networks from the perspective of small-world properties in both normal and psychiatric populations. We found that most of the current results point to a significant trend toward more regularization in normal females and more randomization in normal males in the functional brain networks. On the other hand, there seems to be no consensus to date on the sex differences in small-world properties of the structural brain networks in normal populations. Furthermore, the number of related studies conducted in psychiatric populations is still relatively limited. We anticipate that the conclusions in this manuscript will contribute to a deeper understanding of neurobiological mechanisms underlying sex differences in the brain. Therefore, it is important for future attempts to explore sex differences in memory-specific neural networks, to explore the progressive gender differences of structural brain networks with different weighting approaches, to provide more comprehensive information by using graph analyses with different spatial resolution on the gender related topological differences of structural brain networks, to explore how the gender-related structural brain network differences are associated with the alteration of functional brain networks, and to explore how they evolved over time through simultaneously evaluating the topologies of functional and structural connectivity networks. In addition, determining the distinct pathological mechanisms and the brain sex differences specific to psychiatric populations may help to develop interventions and design innovative treatments better adapted to males and females with this disease. In the future, studies with larger samples and longitudinal designs as well as more studies conducted in psychiatric populations may be warranted to obtain more valuable results.
